# Attraction of Sweet Potato Whitefly, *Bemisia tabaci* (Hemiptera: Aleyrodidae), and Two Generalist Predators to Green Leaf Volatile Compounds

**DOI:** 10.3390/insects15100750

**Published:** 2024-09-28

**Authors:** Alexander M. Gaffke, Neil W. Miller, Anamika Sharma, Sandra A. Allan

**Affiliations:** 1 USDA-ARS, Center for Medical, Agricultural, and Veterinary Entomology, Gainesville, FL 32608, USA; neil.miller@usda.gov (N.W.M.); sandy.allan@usda.gov (S.A.A.); 2 Center for Biological Control, College of Agriculture and Food Sciences, Florida A&M University, Tallahassee, FL 32307, USA; anamika.sharma@famu.edu

**Keywords:** biotype, lure, management, olfactory cues

## Abstract

**Simple Summary:**

The sweet potato whitefly is a major global pest in vegetable production. The management of this pest has typically relied heavily on insecticide treatments. Many populations of sweet potato whitefly are now resistant to insecticides. Therefore, alternative means to control this pest are needed. Many predatory insects consume sweet potato whiteflies, so helping lure and attract both predators and the pest to the same location could improve control. This study investigates attractive odors for sweet potato whiteflies and two predator species that could be used to improve sweet potato whitefly pest management.

**Abstract:**

Traditionally, olfaction was thought to play a minor role in the behavioral ecology of the sweet potato whitefly, *Bemisia tabaci* (Gennadius). However, recent research is uncovering significant potential for whitefly management based on olfaction. Incorporating chemical attractants with standard whitefly management programs could significantly improve control. The integration of attractants with biological control is exceptionally promising. Therefore, the behavioral response of *B. tabaci* and two generalist predators to the green leaf volatiles (*E*)-2-hexenal, (*Z*)-3-hexenal, (*Z*)-3-hexenyl acetate, and (*Z*)-3-hexe-1-ol were investigated in Y-tube olfactometers. Three of the four green leaf volatiles resulted in the attraction of female *B. tabaci*. Blend optimization indicated a two-chemical blend to be the most attractive blend for female *B. tabaci*. In addition, this blend was attractive to female *Macrolophus praeclarus* (Distant) but did not elicit a behavioral response from either male or female *Delphastus catalinae* (Horn). The two-chemical blend of green leaf volatiles could be further developed as a lure to attract *B. tabaci* and its predator, *M. praeclarus*.

## 1. Introduction

The sweet potato whitefly, *Bemisia tabaci* (Gennadius) (Hemiptera: Aleyrodidae), is a polyphagous whitefly with an extremely wide range of host plants [1,2,3] and is known to vector many virus species, including begomovirues, criniviruses, torradoviruses, ipoviruses, and carlaviruses [4]. *Bemisia tabaci* is a major invasive species across the world and is responsible for significant agricultural losses [5,6]. Originally found in and confined to tropical and sub-tropical areas, it quickly spread globally and is now present as a field or greenhouse pest in most countries. Whiteflies can damage plants directly by feeding on their sap and indirectly by vectoring diseases and promoting mold on plant parts due to honeydew production. The most invasive and globally distributed species within this complex are the Mediterranean (MED, known as biotype Q) and the Middle East Asia Minor 1 (MEAM1, known as biotype B).

*Bemisia tabaci,* especially the Q and B biotypes, is under intensive management programs that rely heavily on insecticides, particularly in field settings [7]. These insecticide-heavy management programs have resulted in the rapid development of insecticide-resistant populations of *B. tabaci* [8]. Multiple control strategies have been developed for *B. tabaci* [7], including cultural controls such as mulching [9], sowing dates, and rotational systems [10]; biotechnological strategies [11] and transgenic crops [12]; and biological control with predators, parasitoids, and entomopathogens [7,13]. However, few management strategies have been developed based on the chemical ecology and behavior of whiteflies.

Traditionally, olfaction was considered to play a minor role in *B. tabaci* host selection, with most research focusing on visual preferences [14,15,16]. However, recently, more attention has been paid to the olfactory components of whitefly behavior. Significant interest in developing attractive and repellent compounds for the behavioral manipulation of *B. tabaci* has been expressed, with considerable research devoted to the development of behaviorally active compounds [17,18,19,20]. The repellency of *B. tabaci* has been reported in plant defensive terpenoid compounds [21], such as ocimene and carvacrol [17], limonene [18,20], *p*-cymene and thymol [22], and numerous other unclassified compounds present in plant essential oils [19,23,24]. Attractive compounds from various host plants have also been identified for *B. tabaci* [21], of which two were green leaf volatiles, (*E*)-2-hexenal and (Z)-3-hexen-1-ol [19,20]. Host plant volatiles additionally impact the behavior of natural predators of *B. tabaci*, but the individual chemicals influencing the attraction of natural enemies to *B. tabaci* are understudied [25,26]. Volatile compounds from *Trialeurodes vaporariorum* Westwood (Hemiptera: Aleyrodidae)-infested plants were found to be attractive to the whitefly parasitoid *Encarsia formosa* [27] and a blend of compounds, including (*Z*)-3-hexen-1-ol, were attractive to the parasitoid.

Practical advancements in whitefly management could be achieved through the continued research of *B. tabaci*’s chemical ecology and its integration with biological control programs. Biological control programs developed for *B. tabaci* are promising and can provide significant control of this pest, with some populations being lowered by 90% [28,29]. The natural enemies of *B. tabaci* include species of Hymenoptera, Coleoptera, Hemiptera, Diptera, and Neuroptera, and Arachnida [30]. These invertebrates generally lower whitefly populations by feeding on nymphs and eggs. *Delphastus catalinae* (Horn) (Coleoptera: Coccinellidae), in particular, appears to be a promising component for integrated pest management programs [31,32,33]. This predator has been used extensively in indoor agricultural production for the control of *B. tabaci* as they feed on whitefly eggs and larvae, interrupting the whitefly’s life cycle. While *D. catalinae* is an effective predator of *B. tabaci,* the ability of this insect to persist when *B. tabaci* or alternate prey is not present is currently a limiting factor. Additionally, *D. catalinae* is reported to readily disperse in field settings [34]; therefore, a lure for the attraction of this species to maintain higher densities would be beneficial.

Another emerging natural enemy of interest for controlling *B. tabaci* in the Americas is the native predatory mirid, *Macrolophus praeclarus* (Distant) (Hemiptera: Miridae) [35,36]. *Macrolophus praeclarus* is a zoophytophagous predator, preying on eggs and early instars of various pest species, including the tobacco budworm *Heliothis virescens* and *B. tabaci* [35]. This mirid is widely distributed throughout the Americas and has been studied for its biological traits and potential as a biocontrol agent [36,37]. The zoophytophagous nature of *M. praeclarus* allows for the consumption of target pests when they are present at high densities or plant feeding when prey densities are low. Plant feeding by *M. praeclarus* can induce defensive responses in plants through the upregulation of the jasmonic acid metabolic pathway, resulting in the protection of the plant from piercing and sucking pests [35]. These traits make *M. praeclarus* a promising candidate for biological control programs in varied cropping systems.

Behavioral manipulation, for both pests and beneficials, is an increasingly utilized pest management tactic because of its targeted impact and the low likelihood of developing resistance [38]. This study focuses on the evaluation of promising attractive plant compounds for both *B. tabaci* and two of its predators, *Macrolophus praeclarus* and *Delphastus catalinae,* as a prelude to the development of attraction-based pest management strategies for *B. tabaci* integrated with a biological control and using natural enemies.

## 2. Materials and Methods

### 2.1. Insects

A colony of *B. tabaci* was established from insects collected from greenhouses at the USDA-ARS Laboratory in Tallahassee, FL, USA. They were then reared on potted collard plants (*Brassica oleracea* var. *viridis*) in mesh cages (75 cm × 75 cm × 115 cm, MegaView Science Co., Ltd., Taichung, Taiwan) and maintained at 25 ± 1 °C, 65–75% RH under a photoperiod of 16:8 h (L:D). Insects for use in assays were removed from collard plants and kept in a small plastic holding cage (20 cm × 19 cm × 18 cm), where they were starved for 3 h before testing. Reports from the literature suggest the poor response of males in behavioral assays, and since the larval stage of *B. tabaci* is largely sessile and depends on the oviposition sites selected by females, trials were conducted only with female *B. tabaci* [39].

*Macrolophus praeclarus* was obtained from a colony maintained at the Florida Agricultural and Mechanical University, Tallahassee, Florida. They were reared in cages containing potted tobacco plants (*Nicotiana tabacum*) and fed a supplemental diet of frozen shrimp (*Artemia* spp.) and flour moth (Pyralidae: *Ephestia kuehniella*) eggs (Entofood^®^, Koppert B.V., Veilingweg, The Netherlands). *Delphastus catalinae* were commercially purchased (Evergreen Growers Supply, Clackamas, OR, USA) and maintained on *B. tabaci*-infested collard plants. Both predators were kept in cages in growth chambers at 27 ± 1 °C under a photoperiod of 14:10 (L:D). Before use in assays, individuals of both predator species were collected, sexed, and starved for 3 h. The responses of both sexes of the predators were recorded. Predators were sexed to account for any potential response differences between the sexes and to specifically determine the response of male *M. praeclarus*, as previous research has been focused on female response [40].

### 2.2. Chemicals

Multiple studies have demonstrated the importance of green leaf volatiles to *B. tabaci* behavior [19,20,21]. Additionally, green leaf volatiles are broadly important in plant signaling and attracting natural enemies; therefore, experimentation was focused on the behavioral response towards green leaf volatiles. Synthetic chemicals used in this experiment were purchased from Millipore-Sigma (St. Louis, MO, USA). Four chemicals of varying purity were utilized: (*E*)-2-hexenal (98% purity), (*Z*)-3-hexenal (50%) in triacetin for stabilization, (*Z*)-3-hexenyl acetate (98%), and (*Z*)-3-hexen-1-ol (98%). Compounds were diluted in mineral oil to the various concentrations listed in Table 1. Doses were selected based on preliminary data and reports from the literature [20].

### 2.3. Y-Tube Olfactometer

Bioassays were conducted in a Y-tube olfactometer to measure insect behavioral responses to green-leaf volatiles. The Y-tube was made from clear glass with an internal diameter of 2 cm (Sigma Scientific, Micanopy, FL, USA). The stem of the olfactometer was 13 cm long, and each arm was a length of 8.5 cm. The angle between the arms was 80°. A tank of compressed breathing air equipped with an air pressure regulator (Airgas, Radnor, PA, USA) provided air to the tubing, leading to two branches of a flow meter containing activated carbon filters. The flow meters maintained a flow rate of 0.5 L /min to each arm of the Y-tube. After leaving the meter, air from each meter passed through the tubing to 12.5 mL Erlenmeyer flasks containing distilled water, which acted as a bubbler to humidify the air before it entered a glass chamber (40 cm × 16.5 diam.) used to hold odor sources. One flow meter provided air to one arm of the y-tube and was considered the control arm, and the other meter provided air to the treatment arm. Polytetrafluoroethylene (PTFE) tubing from these chambers connected the airflow to the Y-tube olfactometer, which was positioned flat on the laboratory bench and was surrounded on three sides with white foamcore boards used to standardize visual input to the whiteflies. The overhead light was provided by fluorescent lamps installed above the Y-tube.

Before testing began, chemicals were loaded into 2 µL glass microcapillary tubes (Drummond Scientific Company, Broomall, PA, USA) and sealed on one end with a 1 cm × 1 cm square of Parafilm (Pechiney Plastic Packaging, Menasha, WI, USA). Odor tubes were placed in the designated treatment glass chamber before each round of testing and were allowed to equilibrate for 10 min before experimentation. Bioassays were conducted between 12:00 and 16:00 during a period when whiteflies were consistently responsive (preliminary data, [19,41]). Female whiteflies, identified by the body size and shape of the abdomen [42], were placed individually into the Y-tube stem using a mouth aspirator. Each insect was observed for a maximum of 10 min per trial. A directional choice was recorded when a whitefly moved a minimum of 3.5 cm up an arm of the Y-tube and stayed there for at least 3 s. Insects that did not make a choice after 10 min were recorded as non-responsive. Preliminary trials in the absence of chemical stimulus identified a balanced response of *B. tabaci* to the left (4 individuals) and right (6 individuals) arms of the Y-tube. Trials were replicated 10–20 times per day, with the odor source arriving through one side. Then, the entire apparatus was cleaned with water and detergent (Sparkleen, Fisher Scientific Co., Pittsburg, PA, USA) and dried in a 44 °C laboratory oven overnight (Thermo Fisher Scientific, Waltham, MA, USA). The next day, the olfactometer was reassembled, and replicate bioassays were conducted with treatments on the opposite arm of the y-tube. The orientation of treatment was always changed between days, and all equipment was cleaned when switching between different treatments. Tests were conducted under a 25 ± 2 °C and 50–60% RH, similar to conditions used in other studies [18,19,40,43]. The total replication per experiment ranged between 30 and 56 responding insects.

With female whiteflies, two-choice tests were conducted with the olfactometer comparing three concentrations of single green leaf compounds (*E*)-2-hexenal, (*Z*)-3-hexenal, (*Z*)-3-hexenyl acetate, and (*Z*)-3-hexen-1-ol against controls (Table 1). Based on those results, a 2-chemical blend comprising the two most attractive chemicals was tested against a 3-chemical blend of the three most attractive chemicals. To determine the effect of the combination of the blend of the two most attractive chemicals, it was tested against the most attractive single compound (*Z*)-3-hexenal. The concentrations of single compounds to be tested were initially based on prior published research [20]. Concentrations started at 1.7–1.8 µg and were increased or decreased until attraction was lost, gained, or no behavioral response was detected.

For the evaluation of responses of predators to the same green leaf odor blend, male and female *M. praeclarus,* and *D. catalinae* were tested in the same olfactometer system in a similar configuration with the same environmental conditions. In general, the natural enemies moved more rapidly than whiteflies, and thus, a positive response was recorded for them when they reached the end of an olfactometer arm. Assays with male and female *M. praeclarus* and *D. catalinae* were two-choice assays comparing the most effective attractant for whiteflies, the 2-chemical blend, to purified air. Females of both predators were sourced from mixed colonies and were presumed to be mated and reproductive.

### 2.4. Statistical Analysis

Attraction indices were calculated for each Y-tube paired test using the following formula [20] and are presented in Table 1:

Attraction index = ((Individuals responding towards treatment) − (Individuals responding towards control))/(Total responding).

Then, for each chemical and dose, comparisons were made between those responding to either the treatment or control arm of the Y-tube olfactometer. These analyses were conducted using *χ*^2^ tests with the statistical software R (4.2.2) base package, and comparisons were made between the observed frequencies and the expected frequency of 50:50. The observed frequency was composed of the number of individuals choosing the treatment arm (either a single compound or blend) and the control arm (blank air). Differences with *p*-values < 0.05 were considered significant. Figures display the percentage of responding individuals, which was calculated as the number of individuals responding to the arm of the Y-tube divided by the total number of responding individuals multiplied by 100.

## 3. Results

### 3.1. Response of Whiteflies to Single Compounds

**Figure 1:**
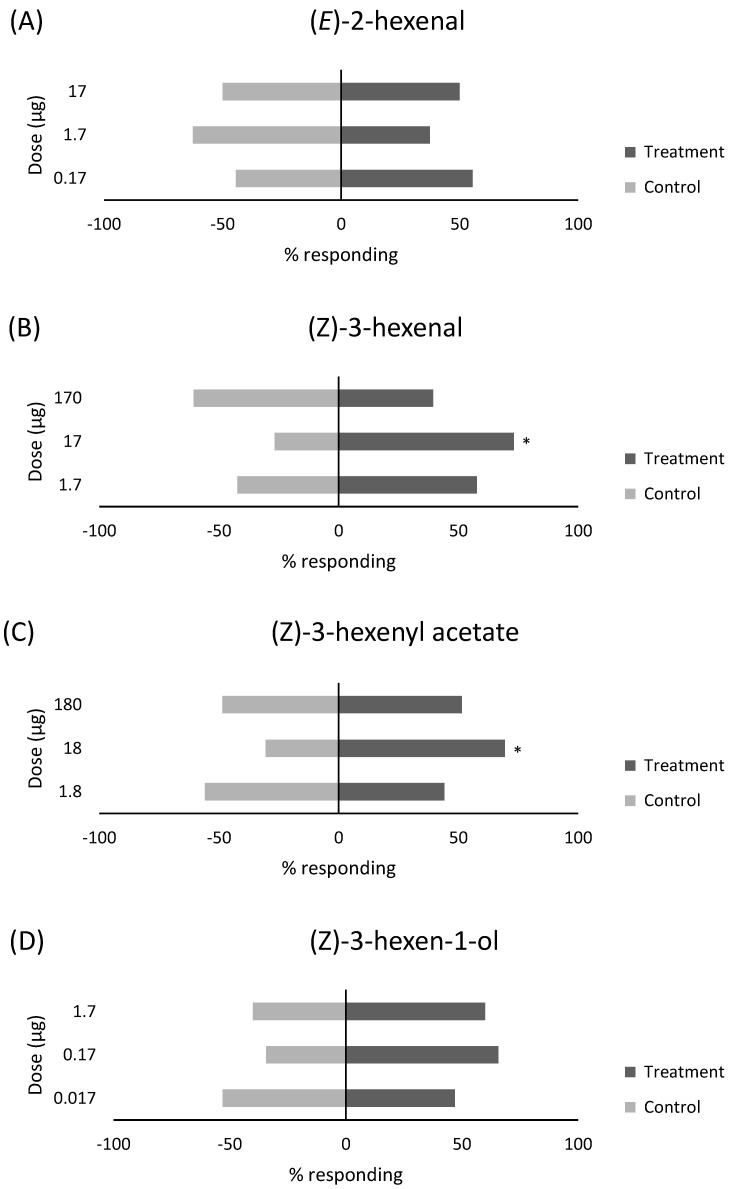
Response of female *B. tabaci* to three doses (µg) of single green leaf chemicals, (**A**) (*E*)-2-hexenal, (**B**) (Z)-3-hexenal, (**C**) (Z)-3-hexenyl acetate, and (**D**) (*Z*)-3-hexen-1-ol (treatment), compared to purified air (control) in a Y-tube olfactometer. * Significant difference from the control (*p* < 0.05, $x^{2}$ test).

The responding number of individuals, non-responding individuals, and attraction indexes of female *B. tabaci* to the single green leaf volatile compounds are listed in Table 1. The behavioral responses of female whiteflies were similar to (*E*)-2-hexenal at the three doses tested (17, 1.7, 0.17 µg) and to the control (Figure 1A), indicating no attraction. At 17 µg, 15 individuals responded to the treatment arm, while 15 individuals went to the control arm, with an attraction index of 0.00 (χ^2^ = 0, d.f. = 1, *p =* 1.0). At 1.7 µg, 12 individuals went to the treatment arm, and 20 went to the control arm, with an attraction index of −0.25 (χ^2^ = 2, d.f. = 1, *p* = 0.15). At the lowest dose 0.17 µg, 20 individuals went to the treatment arm, and 16 individuals went to the control arm, with an attraction index of 0.11 (χ^2^ = 0.44, d.f. = 1, *p* = 0.50).

Female whiteflies exhibited attraction to (*Z*)-3-hexenal at one of the three doses tested, specifically the 17 µg dose (Figure 1B). At the 170 µg dose, 13 individuals went to the treatment, and 20 went to the control arm, with an attraction index of −0.21 (χ^2^ = 1.5, d.f. = 1, *p* = 0.22). For the second dose, 17 µg was attractive to the females, with 41 individuals moving into the treatment arm and 15 choosing the control, for an attraction index of 0.46 (χ^2^ = 12.1, d.f. = 1, *p* < 0.001). The final dose, 1.7 µg, did not elicit a behavioral response, with 30 individuals choosing the treatment arm and 22 selecting the control arm, with an attraction index of 0.15 (χ^2^ = 1.2, d.f. = 1, *p* = 0.26).

Three doses were tested, consisting of 180, 18, and 1.8 µg. One dose of (*Z*)-3-hexenyl acetate was attractive to the female whiteflies (Figure 1C). For the first dose, 18 individuals went to the treatment, and 17 went to the control, with an attraction index of 0.03 (χ^2^ = 0.03, d.f. = 1, *p* = 0.87). The second dose of 18 µg was attractive to the females, with 25 individuals moving into the treatment arm and 11 individuals choosing the control, for an attraction index of 0.39 (χ^2^ = 5.4, d.f. = 1, *p* = 0.02). The final dose did not elicit a behavioral response, with 15 individuals choosing the treatment arm and 19 selecting the control arm, with an attraction index of −0.12 (χ^2^ = 0.47, d.f. = 1, *p* = 0.50).

For (*Z*)-3-hexen-1-ol, there was only slight evidence of attraction to the female whiteflies at one of the three doses evaluated (Figure 1D). At the highest dose of 1.7 µg, 21 individuals went to the treatment, and 14 went to the control arm, with an attraction index of 0.20 (χ^2^ = 1.4, d.f. = 1, *p* = 0.23). The middle dose, 0.17 µg, demonstrated slight evidence of attraction to the females, with 23 individuals moving into the treatment arm and 12 choosing the control, for an attraction index of 0.32 (χ^2^ = 3.5, d.f. = 1, *p* = 0.06). The smallest dose, 0.017, did not elicit a behavioral response, with 23 individuals choosing the treatment arm and 26 selecting the control arm, with an attraction index of −0.06 (χ^2^ = 0.18, d.f. = 1, *p* = 0.66).

### 3.2. Response of Whiteflies to Compound Blends

**Figure 2:**
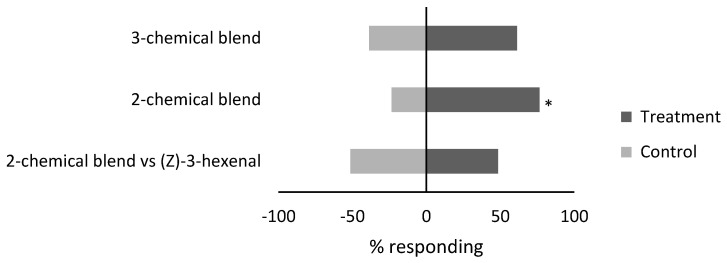
Response (%) of female *B. tabaci* to two and three chemical blends (treatment) compared to purified air (control) and a 2-chemical blend compared to (*Z*)-3-hexenal (control) in a Y-tube olfactometer. Blends tested included a two-component blend of (*Z*)-3-hexenyl acetate (18 µg dose) and (*Z*)-3-hexen-1-ol (0.17 µg dose), and a three-component blend of (*Z*)-3-hexenyl acetate (18 µg dose), (*Z*)-3-hexen-1-ol (0.17 µg dose), and (*Z*)-3-hexenal (17 µg dose). * Significant difference from the control (*p* < 0.05, $x^{2}$ test).

Female *B. tabaci* demonstrated positive attraction towards a two-chemical blend but not the three-chemical blend (Figure 2). The two-chemical blend, consisting of (*Z*)-3-hexenyl acetate (18 µg dose) and (*Z*)-3-hexen-1-ol (0.17 µg dose), attracted 26 individuals, while the control received 8 individuals for an attraction index of 0.53 (χ^2^ = 9.5, d.f. = 1, *p* = 0.002). The three-chemical blend consisting of (*Z*)-3-hexenyl acetate (18 µg), (*Z*)-3-hexen-1-ol (0.17 µg), and (*Z*)-3-hexenal (17 µg) was not attractive, with 19 individuals selecting the treatment arm and 12 individuals selecting the control arm, with an attraction index of 0.22 (χ^2^ = 1.6, d.f. = 1, *p* = 0.21).

In a paired comparison in the Y-tube, there was no difference in the attraction of female *B. tabaci* to a two-chemical blend consisting of (*Z*)-3-hexenyl acetate (18 µg dose) and (*Z*)-3-hexen-1-ol (0.17 µg dose) compared to the single chemical, (*Z*)-3-hexenal (17 µg) (Figure 2). The two-chemical blend attracted 17 individuals, while (*Z*)-3-hexenal attracted 18 individuals for an attraction index of −0.03 (χ^2^ = 0.03, d.f. = 1, *p* = 0.87).

### 3.3. Response of Predators to Compound Blend

**Figure 3:**
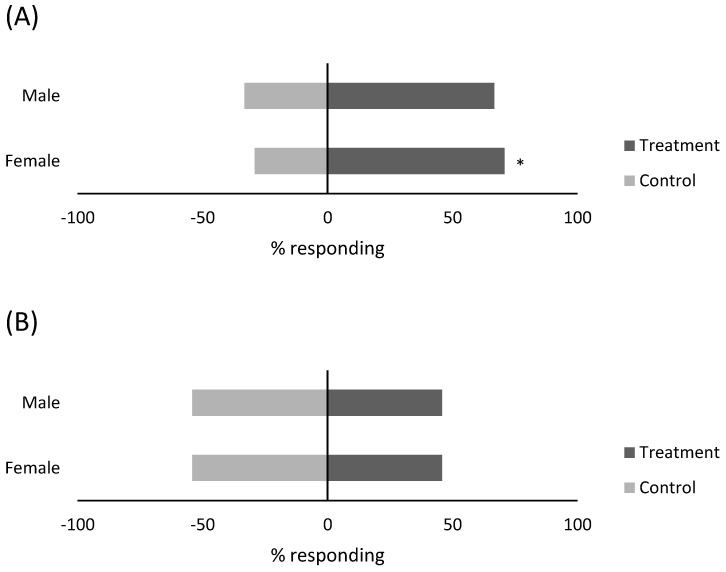
Response (%) of male and female (**A**) *Macrolophus praeclarus* and (**B**) *Delphastus catalinae* to a two-component blend consisting of (*Z*)-3-hexenyl acetate (18 µg dose) and (*Z*)-3-hexen-1-ol (0.17 µg dose) compared to purified air (control) in a y-tube olfactometer. * Significant difference from the control (*p* < 0.05, $x^{2}$ test).

Female *M. praeclarus* demonstrated a positive attraction towards the two-chemical blend, with 17 individuals choosing the treatment arm, 7 choosing the control, and 2 showing no response for an attraction index of 0.42 (χ^2^ = 4.2, d.f. = 1, *p* = 0.04) (Figure 3A). Male *M. praeclarus* did not demonstrate as strong a preference towards the two-chemical blend, with 16 individuals choosing the treatment arm, 8 choosing the control, and 1 with no response for an attraction index of 0.33 (χ^2^ = 2.7, d.f. = 1, *p* = 0.10).

Neither male nor female *D. catalinae* demonstrated a positive attraction towards the two-chemical blend (Figure 3B). For both males and females, 13 individuals chose the control arm, while 11 individuals chose the treatment arm, with an attraction index of −0.08 (χ^2^ = 0.17, d.f. = 1, *p* = 0.68). Males had five no-response individuals, while females had three no-response individuals.

## 4. Discussions

The purpose of this study was to further elucidate the response of *B. tabaci* to common green leaf volatiles, including (*Z*)-3-hexenal, and to develop a blend of compounds that was attractive to whitefly females as well as to two predators, *M. praeclarus* and *D. catalinae. Macrolophus praeclarus* was selected as a target species due to increasing interest in utilizing it as a zoophytophagous biocontrol agent in North America, and *D. catalinae* was selected due to its reputation to be an effective predator of whiteflies and commercial availability [31,33,34,35,36].

This study demonstrates a broader attraction of *B. tabaci* to previously uncharacterized green leaf volatiles, specifically (*Z*)-3-hexenal, and provides contrary evidence to previous studies on the attraction of *B. tabaci* to (*E*)-2-hexenal [20]. (*E*)-2-hexenal, of varying concentrations, is demonstrated to be a strong attractant to *B. tabaci* [20]. However, the results of this study revealed no attraction to (*E*)-2-hexenal. Due to the lack of response at any concentration to this compound, it was not included in the final three-compound blend. The difference in the attraction of *B. tabaci* to (*E*)-2-hexenal between Li et al. (2014) [20] and this study is of note. Attraction towards (*E*)-2-hexenal may be strongly dependent on the physiological state of the insect, mating status, prior exposure, and viral and bacterial load and may possibly vary with local population genetics [44,45,46]. One major difference between the current study and Li et al.’s (2014) [20] is the source plants. Li et al. (2014) [20] sourced *B. tabaci* from *Solanum lycopersicum* L. and did not keep the insects in the colony. The current study sourced *B. tabaci* from *B. oleracea* var. *viridis* and kept them in a colony on these plants. Additionally, Li et al. (2014) [20] utilized wild collected insects. This discrepancy between these two studies highlights the challenges researchers of whitefly chemical ecology face and displays why previously it was believed that semiochemicals played a minor role in whitefly host selection [21].

The single compound with the greatest attraction index for *B. tabaci* was (*Z*)-3-hexenal. However, this single compound was no more attractive than the two-compound blend. In lure development, it is best to select the simplest, most effective mixture of compounds for deployment [47]. This reduces the challenges related to chemical formulation, chemical stability, delivery, and regulatory approval. Thus, a lure consisting only of (*Z*)-3-hexenal would likely be best for real-world applications. However, the cost of (*Z*)-3-hexenal is significantly higher, approximately by a factor of ten, compared to other potential green leaf compounds such as (*Z*)-3-hexenyl acetate and (*Z*)-3-hexen-1-ol. Additionally, (*Z*)-3-hexenal is reactive and requires specialized conditions to be stored [48]. For these reasons, we focused this study on other green leaf compounds and did not test all the possible combinations with (*Z*)-3-hexenal. Under field conditions, it is also expected for (*Z*)-3-hexenal to spontaneously breakdown into (*E*)-2-hexenal, a compound in this study that did not cause attraction [49]. (*Z*)-3-hexenal is also expected to breakdown enzymatically in plants to (*Z*)-3-hexenyl acetate and (*Z*)-3-hexen-1-ol, which is the blend that had the greatest attraction index in this study [49].

Previous studies have shown the attraction of *B. tabaci* parasitoids to green leaf volatiles [26,27]. Our study additionally demonstrates positive chemotaxis of predators to attractive compound blends for *B. tabaci*. Multiple zoophytophagous plant bugs are attracted to the green leaf volatile (*Z*)-3-hexenyl acetate [40], and this study additionally demonstrated the attraction of *M. praeclarus* females to green leaf volatiles. While male *M. praeclarus* did not demonstrate a strong preference for the chemical blend, females did. This lack of a response by males may be beneficial, as cannibalism by male *Macrolophus pygmaeus* (Rambur) (Hemiptera: Miridae) can negatively impact populations [50]. More research is needed to understand the lack of response by male *M. praeclarus*. Other species of *Macrolophus* have males that respond to plant volatiles and females that do not, highlighting that the response may be very species-specific [51]. However, the attraction of females identified in this study could result in increased levels of control by aggregating predators and prey in close proximity. In contrast to the response of *M. praeclarus, D. catalinae* did not respond to the blend. *D. catalinae* may require a more complex blend of compounds to indicate the presence of prey compared to the zoophytophagous *M. praeclarus. Delphastus catalinae* can be attracted to wounded plant material; however, this study demonstrated that this attraction does not likely arise from green leaf volatiles in the two-compound blend [52]. While the chemical blend was not attractive to *D. catalinae,* it did not induce any repellent behavior in *D. catalinae* and could benefit *B. tabaci* biocontrol by aggregating the prey to target areas for the predators. The deployment of these two green leaf volatile compounds will not likely result in an effective attractant to prevent the dispersal of *D. catalinae*.

Results from this study identified attraction behaviors associated with green leaf volatiles in behavioral assays with *B. tabaci*. These results are encouraging, but additional studies examining other plant volatiles as attractants are needed to provide even more attractive compounds. Research in greenhouse and field settings should be conducted to further investigate the response of these insects to tested green leaf volatiles in more complex environments. Additionally, further studies on compound release rates and lure designs will be needed to further investigate the practicality of these compounds to attract whiteflies and their predators.

## Figures and Tables

**Table 1:** Behavioral response of *B. tabaci* females to single green leaf volatile compounds, 3-chemical blends, and a 2-chemical blend (*n* = 30–56).

Compound	CAS Number	Dilution Factor	Dose (µg)	Responses			Attraction Index
				Treatment	Control	No Response	
(*E*)-2-hexenal	1335-39-3	0.01	17	15	15	12	0.00
		0.001	1.7	12	20	8	−0.25
		0.0001	0.17	20	16	4	0.11
(*Z*)-3-hexenal	6789-80-6	0.1	170	13	20	7	−0.21
		0.01	17	41	15	4	0.46
		0.001	1.7	30	22	8	0.15
(*Z*)-3-hexenyl acetate	1708-82-3	0.1	180	18	17	5	0.03
		0.01	18	25	11	4	0.39
		0.001	1.8	15	19	6	−0.12
(*Z*)-3-hexen-1-ol	544-12-7	0.001	1.7	21	14	5	0.2
		0.0001	0.17	23	12	5	0.31
		0.00001	0.017	23	26	11	−0.06
Blend							
3-chemical blend vs. clean air		-	-	19	12	9	0.23
2-chemical blend vs. clean air		-	-	26	8	6	0.53
2-chemical blend vs. (*Z*)-3-hexenal		-	-	17	18	5	−0.03

## Data Availability

Data generated from these experiments is available in the Supplementary Material.

## Supplementary Materials

The following supporting information can be downloaded at https://www.mdpi.com/article/10.3390/insects15100750/s1. File S1: Supplementary data.
